# (*E*)-1-(4-Amino­phen­yl)-3-(pyridin-3-yl)prop-2-en-1-one

**DOI:** 10.1107/S1600536811023634

**Published:** 2011-06-22

**Authors:** Suchada Chantrapromma, Thawanrat Kobkeatthawin, Kullapa Chanawanno, Pitikan Wisitsak, Hoong-Kun Fun

**Affiliations:** a Crystal Materials Research Unit, Department of Chemistry, Faculty of Science, Prince of Songkla University, Hat-Yai, Songkhla 90112, Thailand; b Excellence Center, Mae Fah Luang University, Thasud, Muang, Chaing Rai 57100, Thailand; c X-ray Crystallography Unit, School of Physics, Universiti Sains Malaysia, 11800 USM, Penang, Malaysia

## Abstract

The title chalcone derivative, C_14_H_12_N_2_O, consists of 4-amino­phenyl and pyridine rings bridged by a prop-2-en-1-one unit and exists in a *trans* configuration with respect to the C=C double bond. The mol­ecule is slightly twisted with a dihedral angle of 29.38 (7)° between the benzene and pyridine rings. The prop-2-en-1-one bridge is nearly planar with an r.m.s. deviation of 0.0384 (1) Å and makes dihedral angles of 15.40 (9) and 16.30 (9)°, respectively, with the benzene and pyridine rings. In the crystal, mol­ecules are linked by N—H⋯N and N—H⋯O hydrogen bonds into a layer parallel to the *ab* plane. A π–π inter­action with a centroid–centroid distance of 3.6946 (10) Å is also observed.

## Related literature

For bond-length data, see: Allen *et al.* (1987 ▶). For a related structure, see: Horkaew *et al.* (2010 ▶). For background to and applications of chalcones, see: Gaber *et al.* (2008 ▶); Ávila *et al.* (2008 ▶); Mei *et al.* (2001 ▶); Ohad *et al.* (2004 ▶); Patil *et al.* (2007 ▶); Svetlichny *et al.* (2007 ▶); Tewtrakul *et al.* (2003 ▶); Wu *et al.* (2006 ▶); Xu *et al.* (2005 ▶). For the stability of the temperature controller used in the data collection, see Cosier & Glazer (1986 ▶).
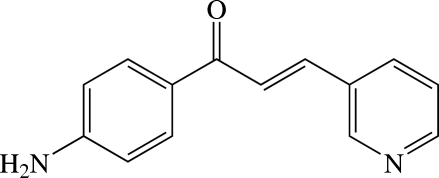


## Experimental

### 

#### Crystal data

C_14_H_12_N_2_O
*M* *_r_* = 224.26
Orthorhombic, 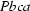

*a* = 12.0046 (12) Å
*b* = 7.9329 (9) Å
*c* = 22.925 (3) Å
*V* = 2183.2 (4) Å^3^
*Z* = 8
Mo *K*α radiation
μ = 0.09 mm^−1^
*T* = 100 K
0.52 × 0.32 × 0.18 mm

#### Data collection

Bruker APEX DUO CCD area-detector diffractometer
Absorption correction: multi-scan (*SADABS*; Bruker, 2009 ▶) *T*_min_ = 0.956, *T*_max_ = 0.985
12726 measured reflections
3177 independent reflections
2433 reflections with *I* > 2σ(*I*)
*R*_int_ = 0.043

#### Refinement

*R*[*F* ^2^ > 2σ(*F* ^2^)] = 0.048
*wR*(*F* ^2^) = 0.132
*S* = 1.03
3177 reflections
202 parameters
All H-atom parameters refined
Δρ_max_ = 0.37 e Å^−3^
Δρ_min_ = −0.18 e Å^−3^

### 

Data collection: *APEX2* (Bruker, 2009 ▶); cell refinement: *SAINT* (Bruker, 2009 ▶); data reduction: *SAINT*; program(s) used to solve structure: *SHELXTL* (Sheldrick, 2008 ▶); program(s) used to refine structure: *SHELXTL*; molecular graphics: *SHELXTL*; software used to prepare material for publication: *SHELXTL* and *PLATON* (Spek, 2009 ▶).

## Supplementary Material

Crystal structure: contains datablock(s) global, I. DOI: 10.1107/S1600536811023634/is2731sup1.cif

Structure factors: contains datablock(s) I. DOI: 10.1107/S1600536811023634/is2731Isup2.hkl

Supplementary material file. DOI: 10.1107/S1600536811023634/is2731Isup3.cml

Additional supplementary materials:  crystallographic information; 3D view; checkCIF report

## Figures and Tables

**Table 1:** Hydrogen-bond geometry (Å, °)

*D*—H⋯*A*	*D*—H	H⋯*A*	*D*⋯*A*	*D*—H⋯*A*
N1—H1*N*1⋯O1^i^	0.88 (2)	2.13 (2)	2.9920 (16)	170 (2)
N1—H2*N*1⋯N2^ii^	0.93 (2)	2.26 (2)	3.1471 (17)	161.7 (19)

Symmetry codes: (i) 
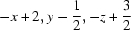
; (ii) 
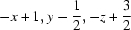
.

**Fig. 1.**
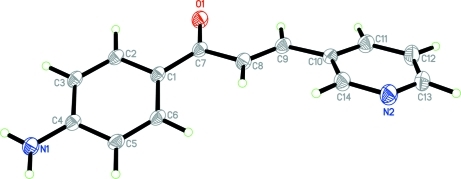
The molecular structure of the title compound, showing 50% probability displacement ellipsoids and the atom-numbering scheme.

**Fig. 2.**
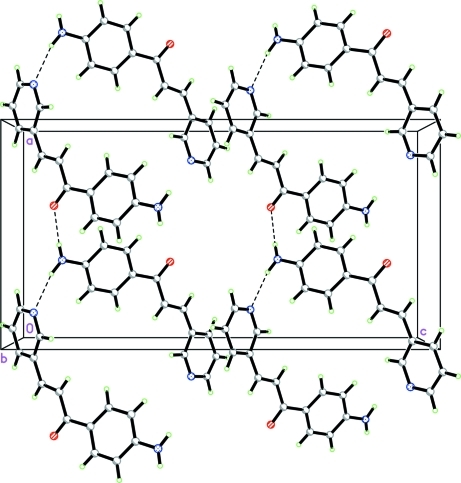
The crystal packing of the title compound viewed along the b axis, Hydrogen bonds were shown as dashed lines.

## supplementary crystallographic information

### Comment

Chalcones are 1,3-diaryl-2-propen-1-ones which can be obtained from both synthetic and natural sources. They have a wide variety of biological activities such as antimalarial (Mei *et al.*, 2001), HIV-1 protease inhibitory (Tewtrakul *et al.*, 2003), antityrosinase (Ohad *et al.*, 2004), antibacterial (Ávila *et al.*, 2008) and antiplasmodial (Wu *et al.*, 2006) properties. Moreover, chalcones have also been studied for non-linear optical (NLO) (Patil *et al.*, 2007) and fluorescent materials (Gaber *et al.*, 2008). These compounds have also been used for sensor, liquid crystal display and fluorescence probe for sensing of DNA or proteins (Svetlichny *et al.*, 2007; Xu *et al.*, 2005). These interesting properties has lead us to synthesize the title compound (I), which contains the amino and pyridine groups in order to study its bioactivity and fluorescent properties. Our results show that (I) was inactive for antibacterial and tyrosinase inhibitory activities. However (I) exhibits weak fluorescence with the maximum emission at 437 nm when was excited at 310 nm. Herein the crystal structure of (I) is reported.

The molecule of the title chalcone derivative (Fig. 1), C_14_H_12_N_2_O, exists in a *E* configuration with respect to the C8═C9 ethenyl bond [1.332 (2) Å] and the torsion angle C7–C8–C9–C10 = -176.57 (13)°. The molecule is twisted with a dihedral angle between the phenyl and pyridine rings being 29.38 (7)°. The prop-2-en-1-one unit (C7-C9/O1) is nearly planar [*r.m.s*. of 0.0384 (1) Å] and the torsion angle O1–C7–C8–C9 being -12.5 (2)°. This middle bridge makes the dihedral angles of 15.40 (9) and 16.30 (9)° with the phenyl and pyridine rings, respectively. The bond distances are of normal values (Allen *et al.*, 1987) and are comparable with the related structure (Horkaew *et al.*, 2010).

In the crystal packing, the molecules are linked by N—H···N and N—H···O hydrogen bonds (Table 1) into sheets parallel to the *ab* plane (Fig. 2). A π–π interaction with a *Cg*1···*Cg*1 distance of 3.6946 (10) Å is observed in the crystal; *Cg*1 is the centroid of the C10–C14/N1 ring. In addition C···C [3.3505 (19) Å; symmetry code 3/2-x, 1/2+y, z and 3.3776 (19) Å: symmetry code 3/2-x, -1/2+y, z], C···O [3.1312 (18) Å; symmetry code 3/2-x, -1/2+y, z] and N···O [2.9920 (16) Å; symmetry code 2-x,1/2+y,3/2-z] short contacts are also observed.

### Experimental

The title compound was synthesized by condensation of 4-aminoacetophenone (0.40 g, 3 mmol) with 3-pyridinecarboxaldehyde (0.18 ml, 3 mmol) in ethanol (15 ml) in the presence of 10% NaOH (aq) (5 ml). After stirring for 2 hr at room temperature, the resulting yellow solid was collected by filtration, washed with distilled diethyl ether, dried and purified by repeated recrysallization from acetone. Yellow block-shaped single crystals of the title compound suitable for *x*-ray structure determination were recrystalized from methanol by the slow evaporation of the solvent at room temperature after several days, Mp. 453-454 K.

### Refinement

All H atoms were located in a difference Fourier map and refined isotropically. The highest residual electron density peak is located at 0.74 Å from C8 and the deepest hole is located at 1.35 Å from C14.

### Crystal data

**Table:**

C_14_H_12_N_2_O	*D*_x_ = 1.365 Mg m^−^^3^
*M**_r_* = 224.26	Melting point = 453–454 K
Orthorhombic, *P**b**c**a*	Mo *K*α radiation, λ = 0.71073 Å
Hall symbol: -P 2ac 2ab	Cell parameters from 3177 reflections
*a* = 12.0046 (12) Å	θ = 2.5–30.0°
*b* = 7.9329 (9) Å	µ = 0.09 mm^−^^1^
*c* = 22.925 (3) Å	*T* = 100 K
*V* = 2183.2 (4) Å^3^	Block, yellow
*Z* = 8	0.52 × 0.32 × 0.18 mm
*F*(000) = 944	

### Data collection

**Table:**

Bruker APEX DUO CCD area-detector diffractometer	3177 independent reflections
Radiation source: sealed tube	2433 reflections with *I* > 2σ(*I*)
graphite	*R*_int_ = 0.043
φ and ω scans	θ_max_ = 30.0°, θ_min_ = 2.5°
Absorption correction: multi-scan (*SADABS*; Bruker, 2009)	*h* = −16→14
*T*_min_ = 0.956, *T*_max_ = 0.985	*k* = −11→10
12726 measured reflections	*l* = −32→23

### Refinement

**Table:**

Refinement on *F*^2^	Primary atom site location: structure-invariant direct methods
Least-squares matrix: full	Secondary atom site location: difference Fourier map
*R*[*F*^2^ > 2σ(*F*^2^)] = 0.048	Hydrogen site location: inferred from neighbouring sites
*wR*(*F*^2^) = 0.132	All H-atom parameters refined
*S* = 1.03	*w* = 1/[σ^2^(*F*_o_^2^) + (0.0702*P*)^2^ + 0.5761*P*] where *P* = (*F*_o_^2^ + 2*F*_c_^2^)/3
3177 reflections	(Δ/σ)_max_ = 0.001
202 parameters	Δρ_max_ = 0.37 e Å^−^^3^
0 restraints	Δρ_min_ = −0.18 e Å^−^^3^

### Special details

**Table:**

Experimental. The crystal was placed in the cold stream of an Oxford Cryosystems Cobra open-flow nitrogen cryostat (Cosier & Glazer, 1986) operating at 100.0 (1) K.
Geometry. All esds (except the esd in the dihedral angle between two l.s. planes) are estimated using the full covariance matrix. The cell esds are taken into account individually in the estimation of esds in distances, angles and torsion angles; correlations between esds in cell parameters are only used when they are defined by crystal symmetry. An approximate (isotropic) treatment of cell esds is used for estimating esds involving l.s. planes.
Refinement. Refinement of F^2^ against ALL reflections. The weighted R-factor wR and goodness of fit S are based on F^2^, conventional R-factors R are based on F, with F set to zero for negative F^2^. The threshold expression of F^2^ > 2sigma(F^2^) is used only for calculating R-factors(gt) etc. and is not relevant to the choice of reflections for refinement. R-factors based on F^2^ are statistically about twice as large as those based on F, and R- factors based on ALL data will be even larger.

### Fractional atomic coordinates and isotropic or equivalent isotropic displacement parameters (Å^2^)

**Table:**

	*x*	*y*	*z*	*U*_iso_*/*U*_eq_	
O1	0.87481 (8)	−0.00829 (13)	0.62116 (4)	0.0274 (2)	
N1	0.88749 (10)	−0.40307 (16)	0.86417 (5)	0.0262 (3)	
H1N1	0.9537 (17)	−0.447 (3)	0.8695 (9)	0.037 (5)*	
H2N1	0.8278 (17)	−0.431 (3)	0.8878 (9)	0.043 (5)*	
N2	0.34284 (10)	0.06758 (18)	0.57106 (5)	0.0317 (3)	
C1	0.82037 (9)	−0.14511 (16)	0.70829 (6)	0.0195 (3)	
C2	0.92774 (10)	−0.20798 (16)	0.72041 (6)	0.0210 (3)	
H2A	0.9864 (14)	−0.193 (2)	0.6922 (7)	0.023 (4)*	
C3	0.95058 (10)	−0.29288 (16)	0.77139 (6)	0.0214 (3)	
H3A	1.0254 (14)	−0.340 (2)	0.7786 (7)	0.029 (4)*	
C4	0.86642 (10)	−0.31820 (17)	0.81357 (6)	0.0208 (3)	
C5	0.75956 (10)	−0.25227 (18)	0.80220 (6)	0.0234 (3)	
H5A	0.7024 (17)	−0.265 (3)	0.8321 (9)	0.045 (5)*	
C6	0.73797 (10)	−0.16943 (17)	0.75074 (6)	0.0214 (3)	
H6A	0.6624 (14)	−0.121 (2)	0.7442 (7)	0.025 (4)*	
C7	0.79831 (10)	−0.05897 (16)	0.65285 (6)	0.0205 (3)	
C8	0.68037 (10)	−0.03488 (18)	0.63425 (6)	0.0226 (3)	
H8A	0.6213 (16)	−0.097 (3)	0.6564 (9)	0.042 (5)*	
C9	0.65435 (10)	0.06655 (17)	0.58997 (6)	0.0232 (3)	
H9A	0.7163 (14)	0.126 (2)	0.5705 (7)	0.031 (4)*	
C10	0.54235 (10)	0.10884 (17)	0.56899 (6)	0.0219 (3)	
C11	0.52786 (11)	0.2397 (2)	0.52922 (6)	0.0284 (3)	
H11A	0.5968 (15)	0.303 (2)	0.5147 (8)	0.030 (4)*	
C12	0.42183 (12)	0.2841 (2)	0.51084 (7)	0.0313 (3)	
H12A	0.4105 (16)	0.374 (3)	0.4827 (9)	0.040 (5)*	
C13	0.33284 (12)	0.1966 (2)	0.53375 (7)	0.0313 (3)	
H13A	0.2553 (16)	0.229 (2)	0.5245 (8)	0.041 (5)*	
C14	0.44619 (11)	0.0255 (2)	0.58784 (6)	0.0267 (3)	
H14A	0.4500 (15)	−0.070 (3)	0.6168 (8)	0.034 (5)*	

### Atomic displacement parameters (Å^2^)

**Table:**

	*U*^11^	*U*^22^	*U*^33^	*U*^12^	*U*^13^	*U*^23^
O1	0.0178 (4)	0.0354 (6)	0.0292 (5)	−0.0004 (4)	0.0009 (4)	0.0037 (4)
N1	0.0177 (5)	0.0342 (7)	0.0267 (6)	0.0028 (4)	−0.0018 (4)	0.0020 (5)
N2	0.0186 (5)	0.0466 (8)	0.0299 (6)	−0.0020 (5)	−0.0015 (5)	0.0051 (5)
C1	0.0148 (5)	0.0193 (6)	0.0245 (6)	0.0002 (4)	−0.0024 (4)	−0.0034 (5)
C2	0.0143 (5)	0.0216 (6)	0.0271 (6)	−0.0001 (4)	0.0004 (5)	−0.0027 (5)
C3	0.0135 (5)	0.0219 (6)	0.0286 (7)	0.0010 (4)	−0.0020 (4)	−0.0031 (5)
C4	0.0169 (5)	0.0219 (6)	0.0237 (6)	0.0004 (4)	−0.0026 (4)	−0.0043 (5)
C5	0.0156 (5)	0.0296 (7)	0.0248 (6)	0.0029 (5)	0.0011 (5)	−0.0027 (5)
C6	0.0142 (5)	0.0243 (6)	0.0255 (6)	0.0023 (4)	−0.0015 (4)	−0.0051 (5)
C7	0.0158 (5)	0.0210 (6)	0.0246 (6)	0.0004 (4)	−0.0013 (4)	−0.0038 (5)
C8	0.0159 (5)	0.0263 (7)	0.0257 (6)	−0.0015 (5)	−0.0013 (5)	−0.0001 (5)
C9	0.0162 (5)	0.0267 (7)	0.0267 (6)	0.0002 (5)	0.0014 (5)	−0.0009 (5)
C10	0.0186 (6)	0.0265 (7)	0.0205 (6)	0.0019 (4)	−0.0003 (4)	−0.0019 (5)
C11	0.0214 (6)	0.0339 (8)	0.0298 (7)	0.0001 (5)	0.0019 (5)	0.0049 (6)
C12	0.0264 (7)	0.0358 (8)	0.0316 (7)	0.0036 (5)	−0.0038 (6)	0.0071 (6)
C13	0.0210 (6)	0.0440 (9)	0.0290 (7)	0.0039 (6)	−0.0043 (5)	0.0027 (6)
C14	0.0190 (6)	0.0348 (8)	0.0262 (7)	−0.0019 (5)	−0.0015 (5)	0.0039 (6)

### Geometric parameters (Å, °)

**Table:**

O1—C7	1.2381 (16)	C5—H5A	0.97 (2)
N1—C4	1.3649 (18)	C6—H6A	0.996 (17)
N1—H1N1	0.88 (2)	C7—C8	1.4909 (17)
N1—H2N1	0.93 (2)	C8—C9	1.332 (2)
N2—C13	1.339 (2)	C8—H8A	1.00 (2)
N2—C14	1.3410 (17)	C9—C10	1.4668 (17)
C1—C6	1.4011 (18)	C9—H9A	0.986 (18)
C1—C2	1.4097 (16)	C10—C11	1.392 (2)
C1—C7	1.4671 (18)	C10—C14	1.3986 (19)
C2—C3	1.3767 (19)	C11—C12	1.3864 (19)
C2—H2A	0.963 (16)	C11—H11A	1.023 (18)
C3—C4	1.4127 (18)	C12—C13	1.378 (2)
C3—H3A	0.988 (17)	C12—H12A	0.97 (2)
C4—C5	1.4097 (17)	C13—H13A	0.99 (2)
C5—C6	1.3749 (19)	C14—H14A	1.01 (2)
			
C4—N1—H1N1	118.9 (13)	O1—C7—C8	119.67 (12)
C4—N1—H2N1	118.2 (12)	C1—C7—C8	118.61 (11)
H1N1—N1—H2N1	121.7 (19)	C9—C8—C7	121.19 (12)
C13—N2—C14	117.14 (13)	C9—C8—H8A	121.1 (11)
C6—C1—C2	117.38 (12)	C7—C8—H8A	117.8 (11)
C6—C1—C7	122.58 (11)	C8—C9—C10	127.07 (12)
C2—C1—C7	120.04 (11)	C8—C9—H9A	117.1 (10)
C3—C2—C1	121.50 (12)	C10—C9—H9A	115.8 (10)
C3—C2—H2A	119.0 (10)	C11—C10—C14	116.83 (12)
C1—C2—H2A	119.5 (10)	C11—C10—C9	119.98 (12)
C2—C3—C4	120.55 (11)	C14—C10—C9	123.18 (12)
C2—C3—H3A	120.7 (10)	C12—C11—C10	120.21 (13)
C4—C3—H3A	118.8 (10)	C12—C11—H11A	121.3 (10)
N1—C4—C5	120.58 (12)	C10—C11—H11A	118.5 (10)
N1—C4—C3	121.28 (11)	C13—C12—C11	117.90 (14)
C5—C4—C3	118.13 (12)	C13—C12—H12A	121.0 (12)
C6—C5—C4	120.50 (12)	C11—C12—H12A	121.1 (12)
C6—C5—H5A	121.3 (12)	N2—C13—C12	123.98 (13)
C4—C5—H5A	118.2 (12)	N2—C13—H13A	115.0 (11)
C5—C6—C1	121.92 (11)	C12—C13—H13A	121.0 (11)
C5—C6—H6A	119.0 (10)	N2—C14—C10	123.89 (14)
C1—C6—H6A	119.0 (10)	N2—C14—H14A	114.7 (10)
O1—C7—C1	121.71 (11)	C10—C14—H14A	121.4 (10)
			
C6—C1—C2—C3	1.23 (18)	O1—C7—C8—C9	−12.5 (2)
C7—C1—C2—C3	−178.45 (12)	C1—C7—C8—C9	168.37 (12)
C1—C2—C3—C4	−0.56 (19)	C7—C8—C9—C10	−176.57 (13)
C2—C3—C4—N1	179.56 (12)	C8—C9—C10—C11	168.40 (14)
C2—C3—C4—C5	−0.85 (19)	C8—C9—C10—C14	−10.3 (2)
N1—C4—C5—C6	−178.84 (13)	C14—C10—C11—C12	1.1 (2)
C3—C4—C5—C6	1.57 (19)	C9—C10—C11—C12	−177.71 (14)
C4—C5—C6—C1	−0.9 (2)	C10—C11—C12—C13	0.9 (2)
C2—C1—C6—C5	−0.50 (19)	C14—N2—C13—C12	1.8 (2)
C7—C1—C6—C5	179.18 (12)	C11—C12—C13—N2	−2.5 (2)
C6—C1—C7—O1	165.18 (12)	C13—N2—C14—C10	0.5 (2)
C2—C1—C7—O1	−15.15 (19)	C11—C10—C14—N2	−1.9 (2)
C6—C1—C7—C8	−15.74 (19)	C9—C10—C14—N2	176.92 (14)
C2—C1—C7—C8	163.92 (12)		

### Hydrogen-bond geometry (Å, °)

**Table:**

*D*—H···*A*	*D*—H	H···*A*	*D*···*A*	*D*—H···*A*
N1—H1N1···O1^i^	0.88 (2)	2.13 (2)	2.9920 (16)	170 (2)
N1—H2N1···N2^ii^	0.93 (2)	2.26 (2)	3.1471 (17)	161.7 (19)

Symmetry codes: (i) −*x*+2, *y*−1/2, −*z*+3/2; (ii) −*x*+1, *y*−1/2, −*z*+3/2.

## References

[bb1] Allen, F. H., Kennard, O., Watson, D. G., Brammer, L., Orpen, A. G. & Taylor, R. (1987). *J. Chem. Soc. Perkin Trans. 2*, pp. S1–19.

[bb2] Ávila, H. P., Smânia, E. de F. A., Delle Monache, F. & Smânia, A. Jr (2008). *Bioorg. Med. Chem* **16**, 9790–9794. 10.1016/j.bmc.2008.09.064 18951808

[bb3] Bruker (2009). *APEX2*, *SAINT* and *SADABS* Bruker AXS Inc., Madison, Wisconsin, USA.

[bb4] Cosier, J. & Glazer, A. M. (1986). *J. Appl. Cryst.* **19**, 105–107.

[bb5] Gaber, M., El-Daly, S. A., Fayed, T. A. & El-Sayed, Y. S. (2008). *J. Opt. Laser Techol* **40**, 528–537.

[bb6] Horkaew, J., Chantrapromma, S., Saewan, N. & Fun, H.-K. (2010). *Acta Cryst.* E**66**, o2346–o2347. 10.1107/S1600536810032514 PMC3007889 21588690

[bb7] Mei, L., Prapon, W. & Mei, L. G. (2001). *J. Med. Chem* **44**, 4443–4452.

[bb8] Ohad, N., Ramadan, M., Soliman, K., Snait, T. & Jacob, V. (2004). *J. Photochem* **65**, 1389–1395.

[bb9] Patil, P. S., Dharmaprakash, S. M., Ramakrishna, K., Fun, H.-K., Kumar, R. S. S. & Narayana Rao, D. (2007). *J. Cryst. Growth*, **303**, 520–524.

[bb10] Sheldrick, G. M. (2008). *Acta Cryst.* A**64**, 112–122. 10.1107/S0108767307043930 18156677

[bb11] Spek, A. L. (2009). *Acta Cryst.* D**65**, 148–155. 10.1107/S090744490804362X PMC2631630 19171970

[bb12] Svetlichny, V. Y., Merola, F., Dobretsov, G. E., Gularyan, S. K. & Syrejshchikova, T. I. (2007). *Chem. Phys. Lipids*, **145**, 13–26. 10.1016/j.chemphyslip.2006.10.001 17125758

[bb13] Tewtrakul, S., Subhadhirasakul, S., Puripattanavong, J. & Panphadung, T. (2003). *Songklanakarin J. Sci. Technol* **25**, 503–508.

[bb14] Wu, X., Tiekink, E. R. T., Kostetski, I., Kocherginsky, N., Tan, A. L. C., Khoo, S. B., Wilairat, P. & Go, M.-L. (2006). *Eur. J. Pharm. Sci* **27**, 175–187. 10.1016/j.ejps.2005.09.007 16269240

[bb15] Xu, Z., Bai, G. & Dong, C. (2005). *Bioorg. Med. Chem* **13**, 5694–5699. 10.1016/j.bmc.2005.06.023 16006133

